# Auxetic Expansion of the Tunica Albuginea for Penile Length and Girth Restoration Without a Graft: A Translational Study

**DOI:** 10.1016/j.esxm.2021.100456

**Published:** 2021-11-06

**Authors:** Alexandre Miranda

**Affiliations:** Reconstructive Urology and Andrology Section, Department of Urology, Ipanema Federal, Rio de Janeiro, Brazil

**Keywords:** Penile Length Loss, Poisson's Rate, Auxetic Technique, 3d Printing, Penile Prosthesis, Peyronie's Disease

## Abstract

**Introduction:**

Several conditions can cause penile length and girth loss. Surgical techniques have been used to restore these penile alterations in patients with severe erectile dysfunction during penile prosthesis implantation. One technique uses multiple small incisions in a mesh pattern (similar to a skin mesh) with satisfactory curvature correction without using a graft, however, this technique does not allow simultaneous increase in penile length and girth.

**Aim:**

To identify a new surgical technique that increases both the length and girth at the same place on the corpora cavernosa (CC), allowing a simultaneously longitudinal and transverse increase of the tunica albuginea.

**Methods:**

A sheet with a star-shaped perforation was created using a mathematical model to allow a longitudinal and transversal increase in the material. Two previously published penile model simulators, with and without deformity, were used to test the mechanical modification of this incision pattern in the CC.

**Main Outcome Measure:**

The effect of the incisions pattern on the geometry of the CC simulator.

**Results:**

The star-shaped incision (auxetic) simultaneously increased the length, girth, and volume of the CC simulator. This auxetic technique could correct any penile deformity, re-establishing the original penile anatomy. The new auxetic incision is only a conceptual and experimental technique awaiting clinical evidence.

**Conclusion:**

The data presented here show that the auxetic technique successfully increases both the length and girth at the same place on the CC simulators, opening a new potential solution to correct challenging cases of complex penile deformities and to restore volume loss.

**Miranda A, Auxetic Expansion of the Tunica Albuginea for Penile Length and Girth Restoration Without a Graft: A Translational Study. Sex Med 2021;9:100456.**

## INTRODUCTION

Penile length and girth loss may be caused by many conditions like Peyronie's disease (PD), postradical prostatectomy, erectile dysfunction following ischemic priapism.^1^, ^2^, ^3^ Some surgical techniques have been created to restore penile length loss (PLL) in patients with severe erectile dysfunction submitted to penile prosthesis implantation (PPI),^4^ including multiple transverse tunica albuginea (TA) incisions without graft, which straightens and lengthens the penis. The procedure was first described in 1995.^5^ In the same year, using the same concept, small incisions in a mesh pattern (similar to a skin mesh) were used to achieve satisfactory curvature correction and restore adequate girth and length.^6^ The author performed transverse incisions on the TA to increase the length of the penis, and longitudinal incisions to increase its girth. In 2020, a report of a mesh technique in a larger number of patients than the previous study revealed good results.^7^ However, the direction of incisions determines the expansion axis. Transverse and longitudinal incisions result in length and girth gains, respectively. Therefore, it is not possible to increase both length and girth at the same place on the corpora cavernosa (CC), a phenomenon that occurs due to mechanical properties of the TA.^8^

This study aimed to address the following question: Is it possible to achieve bidimensional expansion at the same place on the CC, that is, simultaneous longitudinal and transverse increment of the TA, using only incisions without graft? The identification of a new surgical technique with these characteristics could facilitate the 3-dimensional expansion of the CC, with correction of any penile deformity (including the hourglass deformity) and increase in penile girth and length in cases of penile shortening.

## MATERIALS AND METHODS

**Figure 1:**
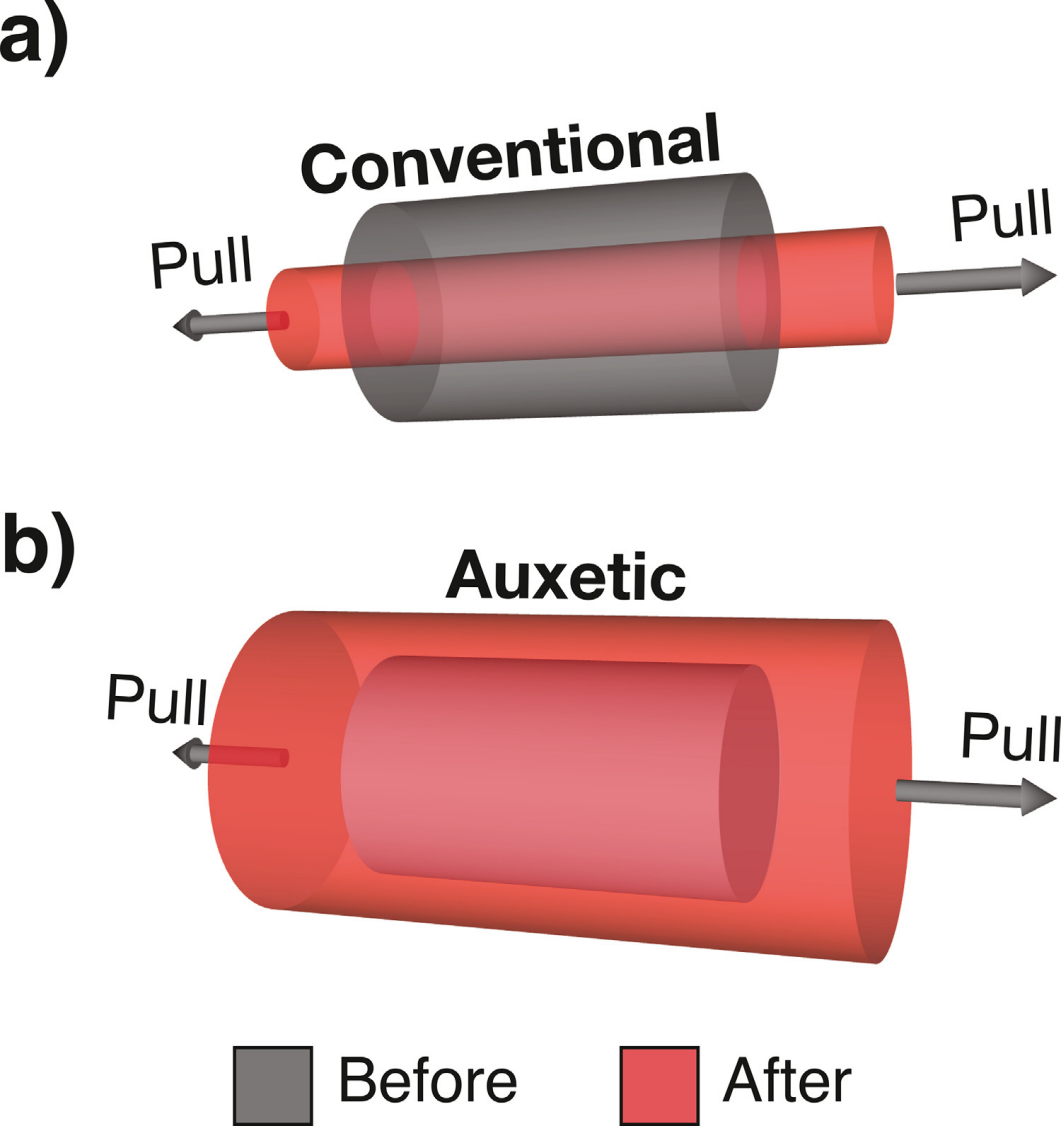
Mechanical property of conventional (a) and auxetic (b) materials. When a conventional material is longitudinally stretched, a reduction in its girth is observed. When an auxetic material is longitudinally stretched, an increase in its girth is observed.

When a material is stretched, it tends to become thinner in the direction perpendicular to that in which it was stretched. The property may easily be visualized by pulling an elastic band, which reveals that the material increases in length and becomes thinner (Figure 1a). This is a fundamental mechanical property of materials and is defined by Poisson's rate (PR). PR is the percentage lateral dimension (width) decrease of a material when stretched, divided by the percentage length increase in direction of stretching. Natural materials, such as the TA, have positive PR values,^8^ which means that stretching the TA will reduce its girth. In 1991, a new type of material with a negative PR value was described. The material possessed a fascinating property in which it became wider when stretched, and was described as auxetic (from the Greek *auxetos* [that may be increased])^9^ (Figure 1b). A negative PR value indicates that a material expands to all directions when pulled in only one.

**Figure 2:**
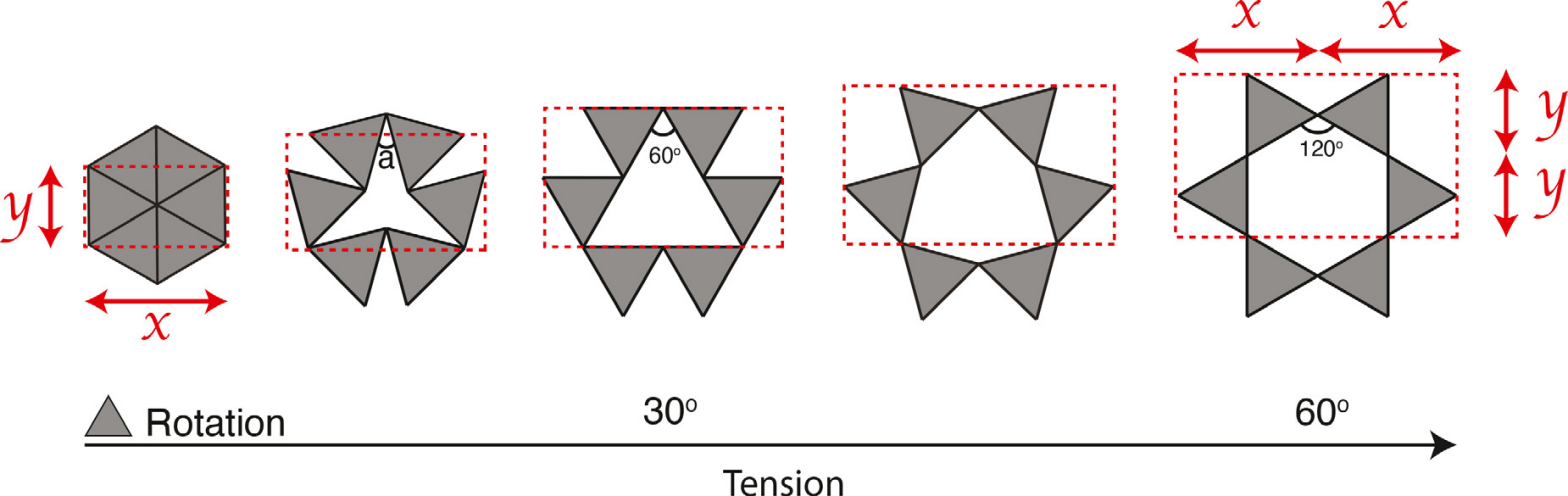
Auxetic behavior created by “rotating rigid equilateral triangles.” The stretching of these systems increases angle "**a**" that occurs between triangles, which produces an open structure, and hence, a negative Poisson's ratio. Note that after rotating triangles 60°, it is possible to double the longitudinal (Y) and transverse (X) dimensions.

In 2000 and 2010, Grima et al. showed that star-shaped perforations produced negative PR values by creating systems that simulated the auxetic “rotating triangles” model.^10^^,^^11^ When the triangles of the system rotated, they caused sheet expansion in 2 dimensions, resulting in tissue increasement that can reach twice the initial size (Figure 2). The finding indicated that the incision pattern could generate negative PR (auxetic) values in the TA.

In order to test the application of this new auxetic incision on the TA, a model study approved by our review board, using 2 previously published penile model simulators,^12^, ^13^, ^14^ was performed to analyze the result of the auxetic incisions in different penile deformities.

A straight penis simulator made of knitted cotton fabric was used to analyze the capacity of the circumferential application of the auxetic incision in increasing the length and girth of the tissue. An analogic caliper was used to measure the length and diameter of the simulators. The girth (circumference) was calculated using the mathematical formula: circumference = (d × π), where d = diameter. The model volume was calculated using the formula to calculate the volume of the cylinder: volume = π × r^2^ × h, where r = radius (diameter/2) and h = height (length).

Previous research showed that alteration of the CC geometry, like the penile aspect ratio (diameter/length) and penile diameter, could modify the penile biomechanical properties.^15^ The Laplace's law [T = p.r, where T = CC wall tension, p = intracavernous pressure, and r = radius (diameter/2)] was used to measure the modification on CC wall tension resulting from the CC incisions. The intracavernous pressure (p) was set at 120 mm Hg in all models. The penile aspect ratio was calculated using the formula d/h, where d = diameter and h = height (length).

Several other penile simulators with different deformities were also created (dorsal curvature, lateral curvature, and dorsal curvature with hourglass deformity [Software: Shapr3D, version 3.23, Hungary] using a 3D printed model.^12^ The model was 3D-printed using a flexible filament of thermoplastic polyurethane with a diameter of 1.75 mm (GTMax3D, Core A3, Brazil) using a fused deposition modeling technique to test the semicircular application of the auxetic incisions for its capacity to correct geometric abnormalities. Finally, the results were illustrated (Adobe Illustrator, version 25.4.1, USA) for better visualization.

## RESULTS

**Figure 3:**
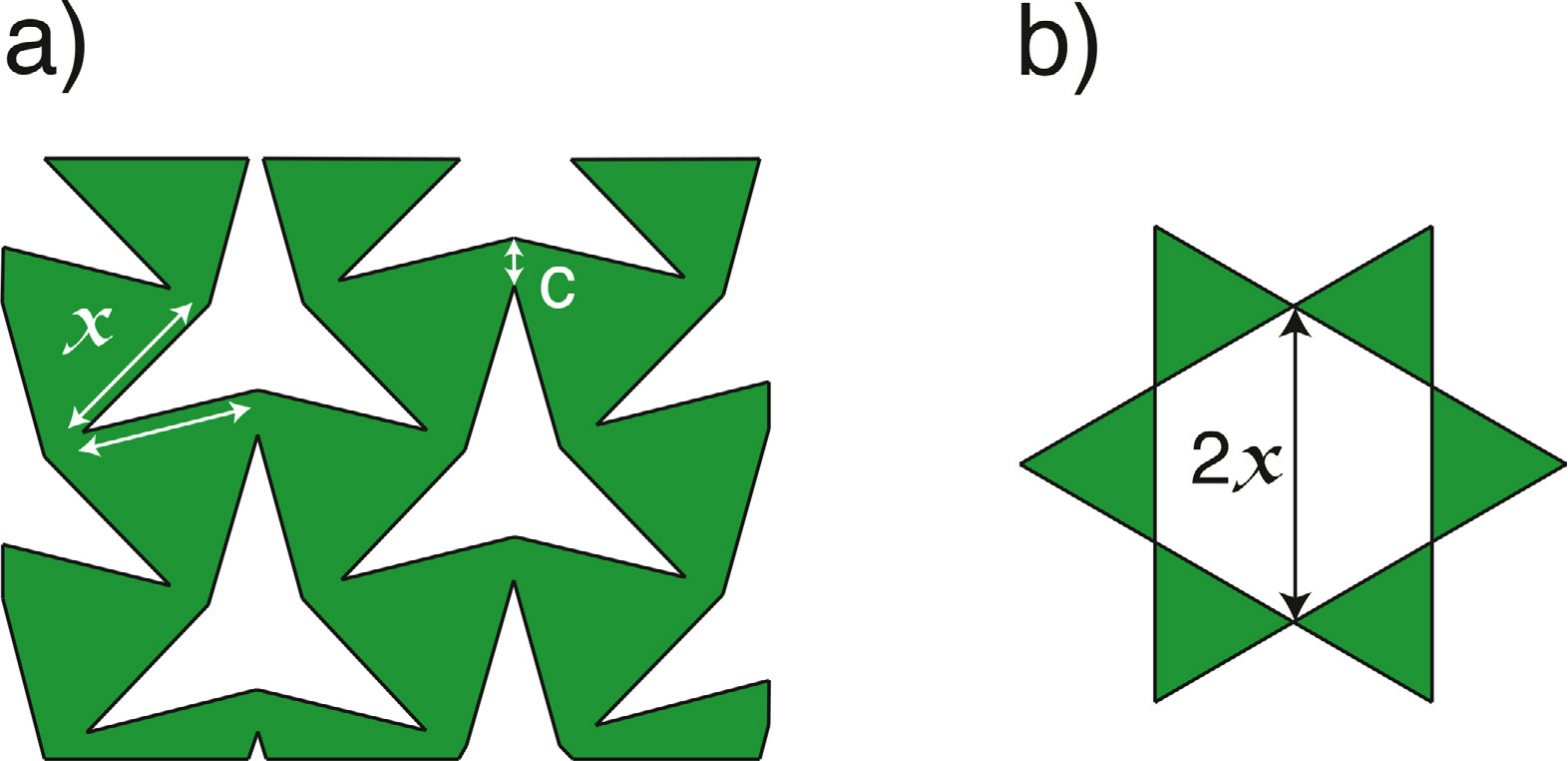
Illustration of the star-shaped incision during tissue expansion. (a) X indicates a linear incision and C is the connection distance between triangles. (b) The maximum diameter of the defect generated after full bidirectional expansion of auxetic tissue is equal to twice the incision size value (2X). The connection size (C) is directly proportional to tissue resistance to tearing, and inversely proportional to tissue expansion due to the restriction of triangle rotation.

Two variables capable of affecting the tissue expansion and defect size of star-shaped incisions were identified. The first variable is connection distance (C) between triangles (Figure 3a). Short C values are unable to overcome tension generated during CC dilation or the insertion of a penile prosthesis [mainly with an inflatable penile prosthesis (IPP) via radial expansion], which results in rupture. Greater C values decrease the degree of triangle rotation, and consequently restrict tissue expansion. The second variable identified was incision size (X) (Figure 3a). The maximum diameter of the defect generated by a star-shaped incision is equal to twice its incision size (2x) (Figure 3b), and should be less than 2 cm long to avoid the need for a graft to repair the defect.^16^

**Figure 4:**
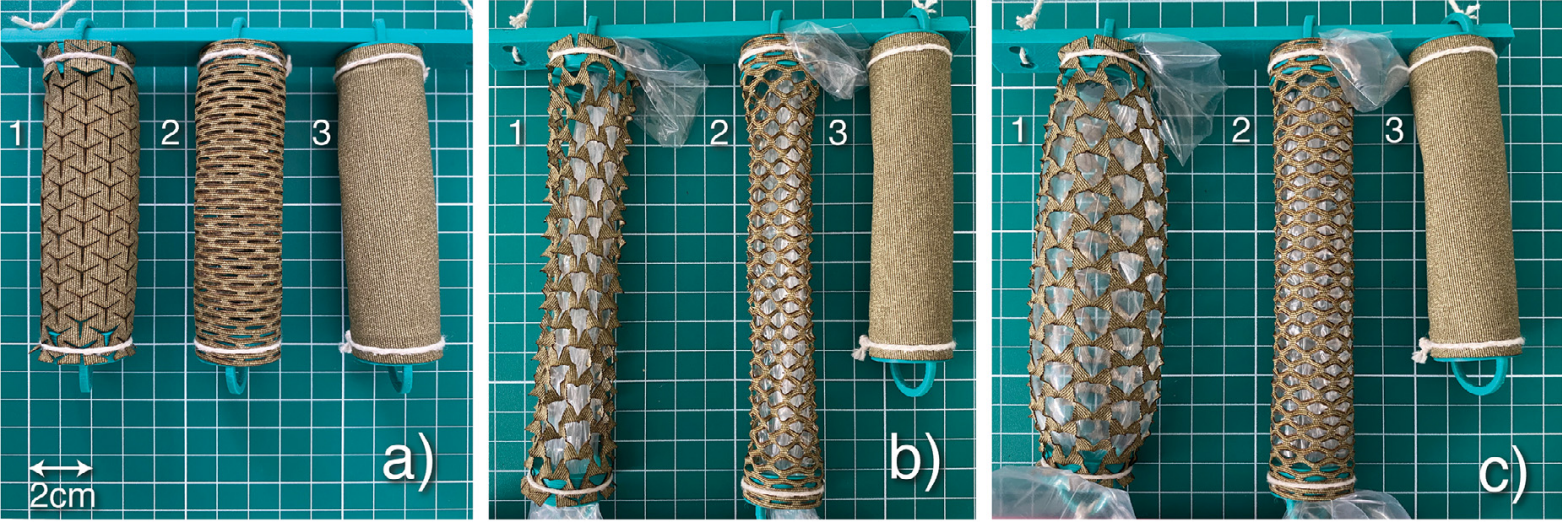
The 3 cylinders used were made of knitted cotton fabric and were each 10-cm long with an 8-cm circumference. Effects of (1) auxetic (star-shaped) and (2) mesh incisions, and (3) no incisions were assessed. (a) Resting state, (b) traction until a length increase of 40% is reached, and (c) an insertion of an inflated balloon inside cylinders with both auxetic and mesh incisions assessed under the same pressure. The control cylinder (3) was not pulled or inflated due to its inelasticity.

**Figure 5:**
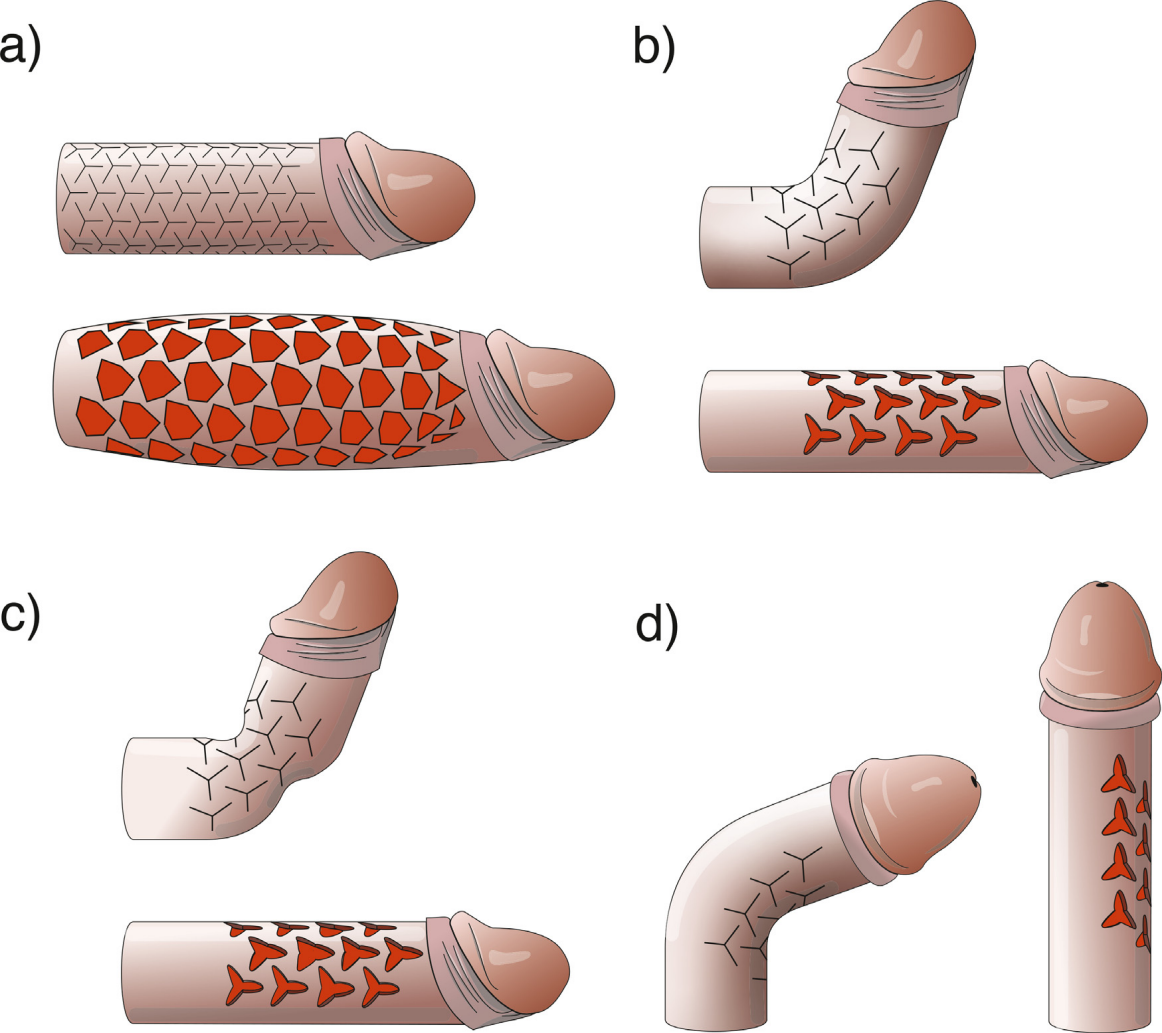
An illustration of an application of the auxetic technique in the corpora cavernosa. (a) Circumferential auxetic incisions simultaneously enhance length and girth. (b) The correction of dorsal curvature using unilateral auxetic incisions is shown. (c) The correction of dorsal curvature with an hourglass deformity using unilateral auxetic incisions is shown. (d) The correction of lateral curvature using unilateral auxetic incisions is shown.

The knitted cotton fabric model,^13^ which simulated a straight CC, was used to test the capacity of the circumferential application of multiple star-shaped incisions to generate auxetic behavior in the TA (simultaneous increase in girth and length). It was compared with the mesh technique (similar to a skin mesh),^7^ because both were made via multiple microincisions (Figure 4a). When the cylinders lengthened by 40%, both incisions (auxetic and mesh) facilitated the longitudinal expansion (from 10 cm to 14 cm) (Figure 4b), but the mesh technique resulted in a girth reduction of 12% (from 8.0 cm to 7.04 cm) (Figure 4c). On the other hand, auxetic incisions resulted in a girth expansion of 50% (from 8.0 cm to 12.0 cm) (Figure 4c, Table 1). Therefore, the use of auxetic incisions around the circumference of the simulated CC simultaneously increased both penile girth and length (Figure 5a). The initial volume of both models was 50.7 cm^3^ (radius (*r*) = 1.27 cm; height (h) = 10 cm). The final volume of the model after stretching and inflation using the mesh technique was 55.17 cm^3^ (*r* = 1,12 cm; h = 14 cm), which corresponded to 108.8% of the initial volume. The auxetic technique resulted in a final volume of 160.45 cm^3^ (*r* = 1,91 cm; h = 14 cm), which corresponded to 316.5% of the initial volume. The final volume obtained using the auxetic technique was 105.28 cm^3^ (2.9-fold) greater than that of the mesh technique (Figure 4c, Table 1).

**Table 1:** Results based on the knitted cotton fabric model

	Baseline	Mesh technique	Auxetic technique	Ratio (auxetic/baseline)	Ratio (mesh/baseline)	Ratio (auxetic/mesh)
Radius	1.27 cm	1.12 cm	1.91 cm	150.4%	88.2%	170.5%
Length	10 cm	14 cm	14 cm	140%	140%	100%
Volume	50.7 cm^3^	55.17 cm^3^	160.45 cm^3^	316.5%	108.8%	290.8%
CC wall tension	1.27 p	1.12 p	1.91 p	150.4%	88.2%	170.5%
penile aspect ratio (d/h)	0.25	0.16	0.27	108%	64%	168.8%

CC = corpora cavernosa; d = diameter; h = length.

The initial tension on the CC wall (T_i_) in all models were the same, where T_i_ = pressure (p) × radius (1.27 cm), that is, T_i_ = 1.27p. The final tension after application of the mesh technique (T_2_) was = pressure (p) x radius_2_ (1.12 cm), that is, T_2_ = 1.12p, and the final tension after application of the auxetic technique (T_3_) was = pressure (p) x radius_3_ (1.91 cm), that is, T_3_ = 1.91p (Table 1).

The penile aspect ratios at the baseline, after mesh technique, and after auxetic technique were 0.25, 0.16, and 0.27, respectively (Table 1).

**Figure 6:**
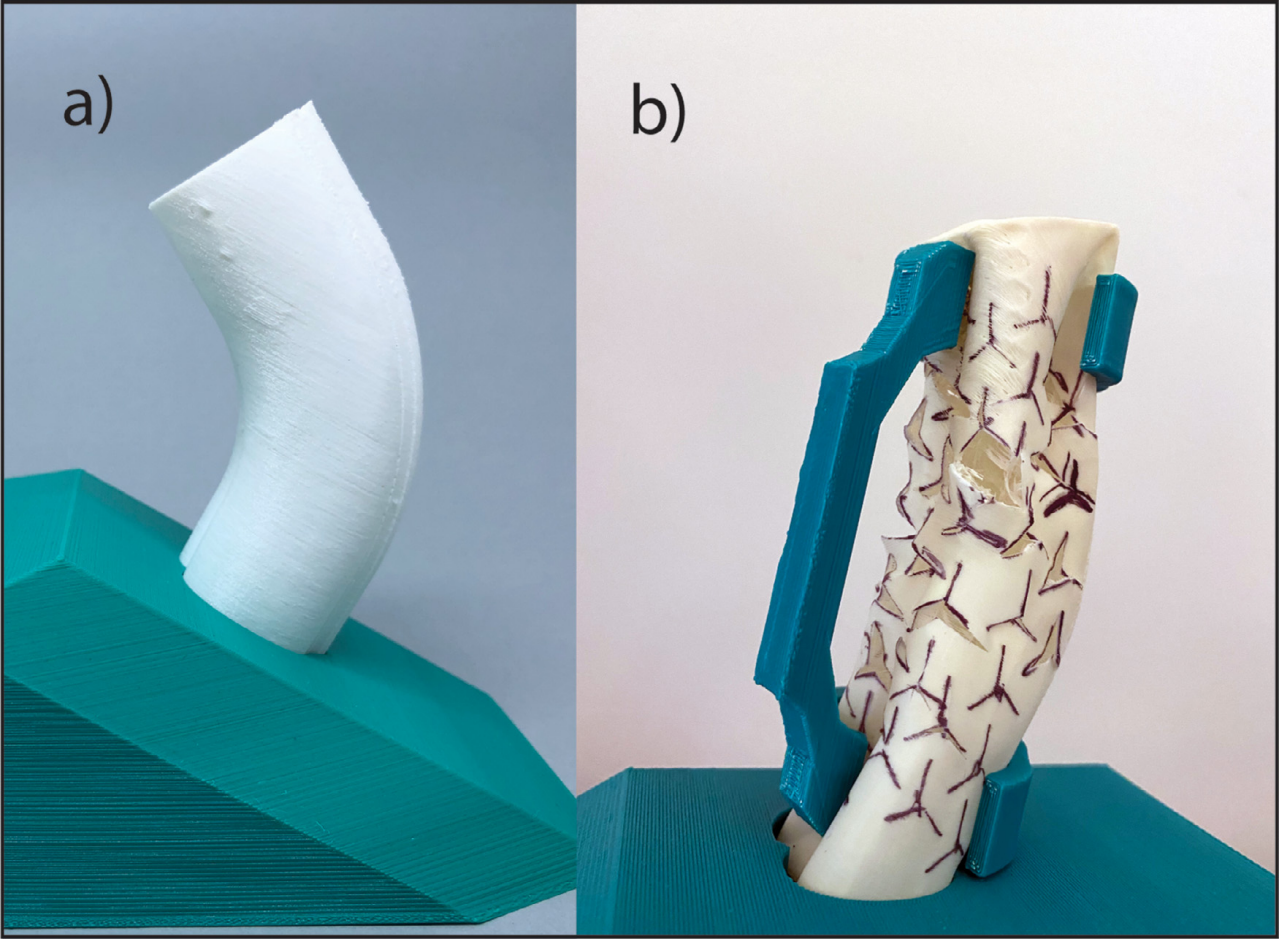
Simulation of penile curvature correction. (a) A 3D printed flexible model with 60° dorsal curvature was created. (b) Auxetic incisions on the short side of penile curvature to rectify the deformity are shown.

The use of auxetic incisions on the short side of penile curvature (semicircular application) in different 3D printed models with a variety of deformities was done. The results illustrated in Figure 5 b-d were based on an individually 3D printed model simulation. One example is shown in Figure 5b, which was based on the 3D printed model presented in Figure 6. The semicircular application of the auxetic incision successfully corrected uniplanar curvature (Figure 5b-d) and curvature with hourglass deformity (Figure 5c) in the 3D printed models.

## DISCUSSION

It is common to receive penile size complaints from patients with PD who had undergone PPI. One reason for this may be that 79.1% of patients with PD experienced subjective PLL that ranged from 0.5 to 7 cm due to PD, and after PPI, 24.1% of these patients reported a mean postoperative subjective PLL of 0.4 cm. After PPI, they reported a significant subjective PLL (mean 3.2 cm) compared with their original penile size. As a result, 82.1% of patients submitted to PPI after PD reported being bothered by the loss of their penile length.^17^

It is clear that during the disease process of PD and treatment with PPI, patients lost a significant proportion of penile length, which resulted in great treatment dissatisfaction. Considering that the mean penile length of healthy individuals is 13.12 cm,^18^ the loss reported^17^ represents an average of 24.4% in penile length. Other authors reported that patients with PD submitted to PPI showed statistically significant reductions in levels of satisfaction, as compared to the general implant population due to final penile dimensions.^19^^,^^20^

The search for a safe, easy, and reliable technique for restoring original penile length and girth is needed for giving patients a treatment that is not only acceptable from the point of view of the surgeon, but also meets patient expectations and restores their capacity to have sexual intercourse in a manner similar to that in which they were accustomed prior to being affected by a penile deformity. The use of auxetic incisions represents a potential solution for this problem since they could simultaneously restore length and girth dimensions without the need for a graft, and facilitate the simultaneous correction of penile curvature, an hourglass deformity, and penile length loss.

The rotation of triangles seen in the knitted cotton fabric model resulted in an excellent degree in tissue expansion, allowing for material (representing the TA) expansion in 2 dimensions, resulting in a cylinder volume (CC simulator) increased to 316.5% (Figure 4c). As a result, the final volume of the auxetic model was 2.9 times larger than that which was produced using the mesh technique. Furthermore, the auxetic technique could correct complex and challenging cases during PPI, including biplanar curvature, and curvature with an hourglass deformity, as seen in Figure 5c. The increased distensibility of tissue generated by the auxetic incisions could facilitates tissue coverage of a penile implant and may potentially be used to correct any deformity type.

It is important to note that it could be possible to create auxetic incisions on only one side of the penis (semicircular application) in cases of curvature correction in which lengthening is not needed, which would avoid the need for making incisions along the entire CC circumference (Figure 5b-d). The partial application of auxetic incisions may reduce the need for detaching the urethra or neurovascular bundle (NVB). In the lateral curvatures, it could be possible only to detach the NVB partially (Figure 5d). If the goal is to increase penile girth, or correct only an hourglass deformity, auxetic incisions may be laterally applied to both sides of the CC without detaching the NVB or urethra. In cases of extensive fibrosis of the CC, such as those that occur post-priapism or penile implant explantation due to an infection, the application of the auxetic technique has the potential to enhance both the diameter and length of the CC due to its capacity to increase tissue distensibility, which provides the space needed to insert a new penile prosthesis and eliminates the need to use a graft to widen the CC.

The mechanically successful coitus is dependent on the penile axial rigidity measured by the penile buckling forces, defined as the magnitude of the axial compressive force (measured in kg), which when applied to the glans of the erect penis results in a pronounced curve such that an additional small force would result in a collapse (buckling) of the erect shaft.^15^ The CC wall tension is one factor that is correlated with axial rigidity.^21^ Comparing 2 inflated cylinders with different diameters but with the same length (h) and internal pressure (p), we found that the CC wall tension in the cylinder with the smaller diameter was reduced. Consequently, a decrease in the cylinder buckling force will be expected, which generates less axial rigidity.^21^ This can be visualized in cases of hourglass deformity. Because of the reduced CC wall tension, the penis bends in the narrow part of the CC when an axial load is applied. The auxetic technique resulted in a 50.4% increase in the CC wall tension in the knitted cotton fabric model, and the mesh technique application a reduction of 11.8% (Table 1). This wall tension difference has an important role in the final penile axial rigidity after the IPP implantation. The IPP, when fully inflated, possesses an internal pressure of approximately 1000 mmHg, independent of the final CC girth.^22^ Therefore, an increase in the CC circumference results in increased axial rigidity.

Another factor related to the penile axial rigidity is the penile aspect ratio (d/h). The auxetic technique increased the d/h ratio by 8%, while the mesh technique reduced it by 36% (Table 1). Thus, all factors modified by the auxetic technique have the potential to increase the final penile axial rigidity.

The application of the auxetic incisions has potential utility for improving surgical technique in other surgical fields in which tissue expansion is needed, such as partial skin graft expansion in extensive burns, difficult abdominal wall closures, and so on.

The new auxetic incision is only a conceptual and experimental technique awaiting clinical evidence. It is important to highlight that the biomechanical propriety of the materials used in the penile models are similar, but not equal to that of TA, constituting a limitation of this study. Future studies will be needed to assess clinical applications of the technique and validate the findings.

## CONCLUSION

Taken together, the findings presented in this study reveal that the auxetic technique was successfully used to confer a negative PR property, increasing both length and girth at the same place on the simulated CC tissue and provided a new potential solution for correcting challenging cases of complex penile deformities and restoring penile volume.
